# Neutrophil membrane biomimetic nanoparticles encapsulating kartogenin promote articular cartilage regeneration

**DOI:** 10.3389/fbioe.2026.1816421

**Published:** 2026-05-08

**Authors:** Zhi Chen, Bingqian Chen, Zhengfei Wang, Fanglu Xu, Hongtao Zhang

**Affiliations:** 1 Department of Orthopedics, The First Affiliated Hospital of Soochow University, Suzhou, China; 2 Department of Orthopedics, Changshu Hospital Affiliated to Soochow University, Changshu No. 1 People’s Hospital, Changshu, China; 3 Department of Nephrology, Changshu No.2 People’s Hospital, Affiliated Changshu Hospital of Nantong University, Changshu, China

**Keywords:** articular cartilage, cell membrane camouflage, inflammation, kartogenin, nanodrug

## Abstract

**Introduction:**

Articular cartilage defect (ACD) is a refractory disease in sports medicine with poor self-healing ability, mainly due to inflammatory infiltration and insufficient chondrogenic differentiation of mesenchymal stem cells (MSCs). Conventional drug delivery systems suffer from poor absorption, rapid immune clearance and low targeting efficiency. Neutrophil membrane biomimetic nanoparticles hold great potential for targeted anti-inflammation and sustained drug delivery, while kartogenin (KGN) is a potent chondrogenic inducer. This study aimed to develop a neutrophil membrane-camouflaged KGN-loaded PLGA nanoparticle (KGN-NNPs) for ACD repair.

**Methods:**

KGN-NNPs were fabricated by wrapping neutrophil membranes onto KGN-loaded PLGA nanoparticles. Physicochemical properties were characterized by TEM, DLS, zeta potential, WB and drug release assays. *In vitro* biocompatibility was evaluated by Live/Dead staining, CCK-8 and cytoskeleton staining. Anti-inflammatory effects were assessed using ELISA, immunofluorescence and qRT-PCR in LPS-induced inflammatory MSCs. Chondrogenic capacity was determined by qRT-PCR, WB and histological staining. A rat ACD model was established to verify *in vivo* cartilage regeneration via macroscopic observation, micro-CT, histological scoring and immunohistochemistry.

**Results:**

KGN-NNPs exhibited uniform spherical morphology with typical core-shell structure, stable size and zeta potential, and retained neutrophil membrane markers (TNF-α R, CD11b). Sustained KGN release over 30 days and pH-responsive release behavior were observed. KGN-NNPs showed excellent biocompatibility without cytotoxicity to MSCs. *In vitro*, KGN-NNPs significantly reduced TNF-α, IL-1β and IL-6 expression and effectively promoted MSCs chondrogenic differentiation by upregulating COL-2, SOX-9 and Aggrecan. *In vivo*, KGN-NNPs remarkably enhanced cartilage and subchondral bone regeneration, with higher ICRS scores, lower Wakitani scores, and increased type II collagen and proteoglycan deposition compared with other groups.

**Discussion:**

Neutrophil membrane-camouflaged KGN-NNPs possess good biocompatibility, targeted anti-inflammatory activity and strong chondrogenic induction ability. This biomimetic nanodrug delivery system significantly promotes articular cartilage regeneration in rat ACD model, providing a novel and promising strategy for the treatment of ACD.

## 1 Introduction

Articular cartilage defect (ACD) is one of the most prevalent conditions in sports medicine clinical practice. Typically caused by traumatic injuries or joint degeneration, it can significantly impair patients’ motor function and overall quality of life (Yoon et al., 2020; Perry et al., 2023; Wesdorp et al., 2022). With the progress of aging population, the incidence of ACD is increasing yearly, bringing heavy burden to the medical expenditure of society (Marchi et al., 2020; Enomoto et al., 2020). Normal articular cartilage consists of chondrocytes and extracellular matrix (ECM), which has good elasticity and play important physiological roles in absorbing shock and reducing friction within the joint. However, due to the lack of blood and lymphatic systems within the cartilage tissue, the proliferation ability of endogenous cells is very poor, thus it is extremely difficult to repair the damaged cartilage (Pan et al., 2022). Intra-articular injection of drugs is a traditional method for the treatment of ACD, commonly used drugs include hormones, antibiotics, and growth factors. However, it has many shortcomings, such as poor drug absorption and easy to be cleared by the body’s immune system, so it is difficult to achieve satisfactory results. Therefore, there is an urgent need to invent safer and more effective drug delivery methods to promote cartilage regeneration in the field of ACD treatment.

The emergence and development of nanodrug delivery technology (NDDT) has brought hope for solving the above problems. Compared with traditional drugs, nanodrugs are typically designed for a smaller size, which makes them more soluble and easier to pass through the physiological barriers of the body (Zhao et al., 2022; Mi et al., 2021). Additionally, NDDT can also greatly help to improve the stability of drugs, increase the local concentration of drugs, and reduce adverse drug reactions (Hu et al., 2023). The delivery of nanodrugs depend on specific drug carriers, commonly used drug carriers include polymers, micelles, and liposomes. Poly (lactic-co-glycolic acid) (PLGA) is a kind of high polymer with good biocompatibility and degradability, which is suitable for encapsulation and delivery of drug molecules. In addition, the release rate of drugs can be controlled by adjusting the size, surface properties of the nanoparticles (NPs) (Li et al., 2022). However, under the influence of the complex immune microenvironment in the body, NDDT still has certain disadvantages such as poor drug targeting and easy immune phagocytosis, which are urgent problems to be solved in the current field (Huang et al., 2024; Zhao et al., 2021).

Cell membrane biomimetic technology (CMBT) is a novel biotechnology that has emerged in recent years. By wrapping different cell membranes on the surface of NPs, specific biological functions can be achieved (Ijaz et al., 2024; Wang Z. et al., 2023; Nica et al., 2023). For example, erythrocyte membrane can reduce immunogenicity and avoid phagocytosis by macrophages; neutrophil membrane can enhance the chemotaxis of nanodrugs to inflammatory sites and play an targeted anti-inflammatory effect; cancer cell membrane can achieve immune escape and enhance the targeting of nanodrugs to tumors (Ren et al., 2024; Lin et al., 2023; Li et al., 2023; Huang et al., 2023). By combining CMBT with NDDT, the targeting capacity of nanodrugs can be improved, making it more precise in delivering its therapeutic effects. Meanwhile, it can enhance the biocompatibility of nanodrugs and reduce its immunogenicity, thereby prolonging the duration of nanodrugs in the body (Zhu et al., 2021). The combination of CMBT and NDDT amplifies the advantages of nanodrugs and overcomes the disadvantages, thus has become a promising approach in the field of nanomaterials.

Inflammation plays an important role in the progression of ACD. Cartilage damage can release inflammatory factors and damage-associated molecular patterns (DAMPs) to trigger an inflammatory response, eventually lead to the apoptosis of chondrocytes (Li et al., 2020). The activation of DAMPs will recruit immune cells and inflammatory factors such as interleukin-1 (IL-1), interleukin-6 (IL-6), and tumor necrosis factor-α (TNF-α), further exacerbating cartilage damage (Feng et al., 2022; Rai et al., 2021). Cartilage damage and inflammatory response mutually reinforce each other, forming a vicious cycle, ultimately leading to cartilage degeneration and joint dysfunction. Meanwhile, the high level of inflammatory factors seriously impedes the differentiation of mesenchymal stem cells (MSCs) into chondrocytes, hindering the generation of new cartilage (Hossain et al., 2023). Furthermore, the inflammatory response activates oxidative stress, producing large amounts of reactive oxygen species and nitrogen oxides (Shen et al., 2023; Sadri et al., 2023). Blocking the activation of inflammatory factors or regulating their balance can help reduce cartilage damage and improve joint function. Neutrophil membrane-coated NPs have a natural chemotactic property towards inflammation signals due to the retention of inflammation-related receptors on the outer layer of the NPs (Liu et al., 2023). After being released and reaching the inflammation site, neutrophil membrane-coated NPs can neutralize inflammatory reactions, reducing the damage to cells caused by inflammatory factors. In addition, they can inhibit the recruitment of macrophages to the inflammation site, thereby alleviating the inflammatory response and providing a favorable environment for tissue repair (Huang et al., 2022). Applying neutrophil membrane-coated NPs to the treatment of ACD may be a novel and promising strategy.

**FIGURE 1:**
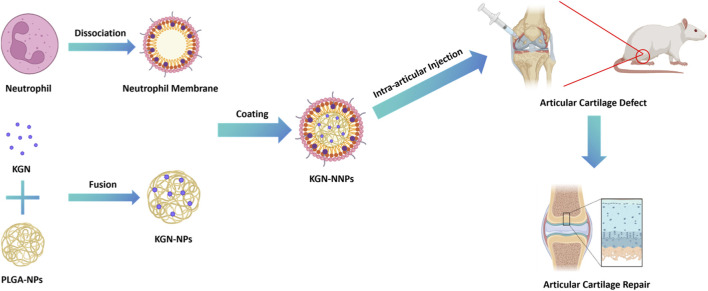
Schematic diagram of the preparation process and application of KGN-NNPs in cartilage regeneration. The cell membrane of neutrophil was stripped and wrapped on the surface of KGN-NPs to prepare KGN-NNPs. The synthesized KGN-NNPs were intra-articular injected into the defect area of the rat ACD model to obtain the subsequent cartilage repair effect.

The formation of chondrocytes rely on the stimulation of MSCs by specific bioactive factors. Kartogenin (KGN) is a small heterocyclic compound that was discovered previously, which was proved has a strong ability to induce MSCs to differentiate into chondrocytes. It has got recognized and has been preliminarily applied in cartilage tissue engineering (Hou et al., 2021; Xu et al., 2021). However, natural KGN has disadvantages including poor solubility, low drug concentration, short half-life, fast drug release, and strong immunogenicity, which greatly limit its further application (Wytrwal et al., 2023). How to overcome these drawbacks has become an urgent matter to be solved in the current research field. In order to improve the delivery mode and bioavailability of KGN, we designed and synthesized a cell membrane biomimetic nanoparticle with an outer layer of neutrophil membrane, an inner layer of PLGA NPs, and a core of KGN in this present study (Figure 1). The synthesized composite nanoparticles (named KGN-NNPs) not only can greatly enhance the solubility of KGN, improve its biocompatibility, but also can prolong its release time and increase the drug concentration. Furthermore, benefit from the camouflage of neutrophil membrane, the KGN-NNPs are expected to have immune evasion ability, avoiding being cleared by immune system, and target adsorption to inflammatory factors to reduce inflammation reaction. The application of this biomimetic nanodrug delivery system is expected to provide a new strategy for the clinical treatment of ACD.

## 2 Materials and methods

### 2.1 Materials

KGN and PLGA were purchased from Sigma-Aldrich Inc. (St. Louis, United States). Lipopolysaccharide (LPS) was purchased from PeproTech (New Jersey, United States). Live/Dead assay kit was purchased from Invitrogen (Carlsbad, United States). Rhodamine-Phalloidin, DAPI and enzyme-linked immunosorbent assay (ELISA) kit were purchased from Beyotime Biotechnology (Shanghai, China). Cell counting kit-8 (CCK-8) was purchased from Dojindo Molecular Technologies Inc. (Kumamoto, Japan). Alcian blue staining kit, safranin-O staining kit, toluidine blue staining kit were purchased from Solarbio Life Sciences (Beijing, China). Antibodies were purchased from Abcam (Cambridge, United Kingdom). Primers were was purchased from Sangon Biotech (Shanghai, China).

### 2.2 Preparation of KGN-NNPs

Firstly, 100 mg KGN and 100 mg poly (lactic-co-glycolic acid)-polyethylene glycol-carboxyl (PLGA-PEG-COOH) were dissolved with 5 mL chloroform to prepare 2% polyvinyl alcohol (PVA) solution. After ultrasonic reaction for 5 min, the mixed solution was transferred to a three-neck flask and mechanical stirring for 4 h, then washed three times with ultrafiltration tube and concentrated to prepare KGN@PLGA. In order to prepare KGN-NNPs, neutrophils were extracted from the peripheral blood of the Sprague-Dawley (SD) rats, then cracked by 0.25 mM ethylenediamine tetraacetic acid (EDTA) with protease inhibitors (Beyotime, Shanghai, China) at 4 °C for 1 h, followed by an enucleating process using a homogenizer with a rotation speed of 2,800 rpm for 20 times. The vesicles (neutrophil membranes) were collected after centrifuging with an ultracentrifuge at 4 °C for 1 h. Next, the extracted neutrophil membrane and KGN@PLGA were fully dispersed in phosphate buffer solution (PBS), and ultrasonicated for 30 min. Finally, the mixture was squeezed back and forth 10 times through 400 and 200 mesh screens by a liposome extruder to prepare KGN-NNPs.

### 2.3 Characterization of KGN-NNPs

A transmission electron microscope (TEM, FEI, United States) was used to observe the morphology characteristics of KGN-NNPs. The particle size and zeta potential of KGN-NNPs were measured by a Zetasizer analyzer (Malvern Panalytical, United Kingdom) after suspending in deionized water and PBS. The stability of KGN-NNPs in different media was also evaluated by dynamic light scattering (DLS). The encapsulation efficiency (EE %) and drug loading capacity (DLC %) of the KGN@PLGA were determined with NanoDrop 2000 spectrophotometer (Thermo Fisher Scientific, Waltham, United States) using the following formulas: *EE %* $=\frac{W_{total}-W_{free}}{W_{total}}\times 100\;\%$ *, DLC %* $=\frac{W_{total}-W_{free}}{W_{NP}}\times 100\;\%$ *,* where *W*_*total*_ represents the total weight of the drug initially added, *W*_*free*_ denotes the weight of the free drug in the supernatant after ultracentrifugation at 10,000 rpm for 15 min, and *W*_*NP*_ is the total weight of the harvested NPs. The release of KGN was determined by dialysis method. In brief, KGN-NNPs were suspended with PBS in a dialysis bag and incubated in 250 mL PBS (pH = 7.4, 6.5) at 37 °C with a rotation speed of 120 rpm. Then the absorption spectrum of KGN was detected at particular time points with a NanoDrop 2000 spectrophotometer (Thermo Fisher Scientific, Waltham, United States). The expression of receptor proteins in the surface of KGN-NNPs were determined by Western blot (WB). To be specific, total proteins of neutrophil, vesicles and KGN-NNPs were extracted with RIPA lysis buffer (Beyotime, Shanghai, China), followed by quantifying total proteins with a BCA protein assay kit (Beyotime, Shanghai, China). Then the protein samples were transferred to a polyvinylidene difluoride (PVDF) membrane after electrophoresis with a SDS-PAGE gel. Primary antibodies of TNF-α receptor (TNF-α R) and CD11b (Abcam, Cambridge, United Kingdom) were incubated overnight with the samples at 4 °C. The images were detected through an electrochemiluminescence reagent autoradiography (Bio-Rad, Hercules, CA, United States) after incubation with horseradish peroxide (HRP)-labeled secondary antibodies.

### 2.4 Isolation and culture of MSCs

Primary bone marrow MSCs were isolated from the femurs of 4-week-old SD rats by direct adherence method as previously described (Maridas et al., 2018). The Minimum Essential Medium Eagle-Alpha Modification (Alpha MEM, Hyclone, UT, United States) with 1% penicillin-streptomycin (PS, Gibco®, Life Technologies Pty Ltd., Australia) and 10% fetal bovine serum (FBS, Hyclone, UT, United States) was used for cell culture. Cells were cultured in an incubator at 37 °C and 5% CO_2_ atmosphere, and the cell culture medium was first changed at 24 h and then changed every 48 h. When adherent cells reached 90% confluence, they were passaged using 0.25% Trypsin-EDTA (Sigma-Aldrich, St. Louis, United States). Cells from passage P2 and later were used for the following experiments.

### 2.5 Biocompatibility evaluation of KGN-NNPs

In order to evaluate the safety of the KGN-NNPs, MSCs were cultured with KGN solution (0.1 mg/mL) and KGN-NNPs respectively, then Live/Dead staining was performed on day 1 and day 5 to observe the cell viability. In brief, after removing the culture medium, the Live/Dead staining kit was added into the wells and incubated at 37 °C for 30 min, then a fluorescence microscope (Thermo Fisher Scientific, Waltham, MA, United States) was used for observing cell survival status and calculating number and ratio of viable cells. To better assess cell morphology, cytoskeleton staining was performed at 3 days after culturing. After being fixed with 4% paraformaldehyde, permeabilized with 0.3% Triton X-100, and blocked with 3% bovine serum albumin (BSA) for 1 h, the F-actin of the samples were stained with Rhodamine-Phalloidin for 40 min, the nucleus was stained with DAPI for 10 min, finally observed and photographed under a fluorescence microscope. Meanwhile, CCK-8 assay was conducted at day 1, day 3 and day 5 after co-culturing to evaluate cell proliferation. Reagents were added to each well and incubated in the dark at 37 °C for 2 h, and then the absornance at 450 nm was measured with a multimode reader (Thermo Fisher Scientific, Waltham, United States).

### 2.6 *In vitro* anti-inflammation evaluation

MSCs (2 × 10^4^ cells/well) were cultured with KGN (0.1 mg/mL) and KGN-NNPs respectively in order to verify the *in vitro* anti-inflammation capacity of the NPs. When the cells were completely adherent, 200 ng/mL LPS was added to the culture medium to induce inflammatory response for 24 h, then the culture medium was first changed and followed by change every 48 h. The group without LPS was labeled as Negative Control (NC) group; the group added LPS was labeled as Positive Control (PC) group. On day 3 and day 7 after induction, the levels of TNF-α and IL-1β in the culture system were measured with ELISA. To be specific, the culture supernatants were collected and immediately centrifuged at 3,000×g at 4 °C for 10 min. Next, the supernatants were transferred to an ultrafiltration centrifuge tube with a molecular weight cutoff of 100 kDa and centrifuged again at 4,000×g at 4 °C for 15 min to remove residual NPs. Finally, the levels of inflammatory factors in the supernatants were determined using ELISA kits according to the manufacturer’s instructions.

Immunofluorescence staining was also used to evaluate the anti-inflammation capability of KGN-NNPs. After 7 days of culturing, the culture medium was discarded, and the cells were fixed with 4% paraformaldehyde, permeabilized with 0.3% Triton-X100, and blocked with 3% BSA. Then, the cells were incubated overnight with primary antibodies of TNF-α and IL-1β respectively, and followed by incubating with secondary antibodies for 2 h at room temperature. After staining with TRITC, FITC-phalloidin and DAPI, the images were captured using a fluorescence microscope. The normalized immunofluorescence intensity measured by ImageJ software was also acquired for semi-quantitative analysis of the expression of relevant inflammatory factor receptors.

After co-culturing for 7 days, Quantitative Real-Time Reverse Transcription PCR (qRT-PCR) was applied to detect the gene expression of TNF-α, IL-1β and IL-6. The relevant gene sequences were listed in Table 1. Firstly, total mRNA was extracted from the cells using TRIzol reagent (Invitrogen, California, United States), then the amount of mRNA was determined by NanoDrop 2000 spectrophotometers (Thermo Fisher Scientific, MA, United States). Afterwards, the mRNA was reverse-transcribed into cDNA using RT Master Mix (ABM, Vancouver, Canada). In detail, The mixture was prepared by adding 1 μl of primer, 1.5 μl of nuclease-free water, 2.5 μl of cDNA, and 5 μl of Master Mix to a total volume of 10 μl for use in qRT-PCR. The gene expression levels were quantified through SYBR GreenMaster (Bio-Rad, Hercules, CA, United States). The gene amplification procedure was performed according to the following steps: 95 °C for 30 s to pre-denaturation, 40 cycles at 95 °C for 5 s to denaturation, 60 °C for 30 s to annealing and extension. The relative expression levels were calculated by 2^-△△Ct^ Method and normalized to Gapdh.

**TABLE 1:** Primers for qRT-PCR.

Target gene	Forward	Reverse
Gapdh	GCAAGTTCAACGGCACAG	CGC​CAG​TAG​ACT​CCA​CGA​C
TNF-α	GGC​GTG​TTC​ATC​CGT​TCT​CTA​C	ACT​TCA​GCG​TCT​CGT​GTG​TTT​C
IL-1β	TTC​AAA​TCT​CAC​AGC​AGC​ATC​TCG	ACA​CTA​GCA​GGT​CGT​CAT​CAT​CC
IL-6	CTT​CCA​GCC​AGT​TGC​CTT​CTT​G	TGG​TCT​GTT​GTG​GGT​GGT​ATC​C
COL-2	GGA​GCA​GCA​AGA​GCA​AGG​AGA​AG	TCA​GTG​GAC​AGT​AGA​CGG​AGG​AAA​G
SOX-9	GCT​TGA​CGT​GTG​GCT​TGT​TC	GAG​CCG​GAT​CTG​AAG​ATG​GA
Aggrecan	AAT​CCA​GAA​CCT​TCG​CTC​CAA​TGA​C	GGT​GGC​TTC​GCT​GTC​CTC​AAT​G

### 2.7 *In vitro* chondrogenic assessment

qRT-PCR was used for determining the expression of chondrogenic genes. After co-culturing for 7 days, the relative mRNA levels of COL-2, SOX-9, and Aggrecan were detected by the same protocol as described above. All the relevant gene sequences were listed in Table 1. WB was applied to further determine the expression of chondrogenic related proteins in each group after 7 days of culturing. To be specific, firstly, total proteins were extracted from cells with RIPA lysis buffer (Beyotime, Shanghai, China) under the protection of protease inhibitor. Then protein concentration was quantified with BCA protein quantification kit (Beyotime, Shanghai, China). Afterwards, 25 μg of protein was separated by SDS-PAGE gel from each well and transferred to a PVDF membrane. After blocking with 5% BSA, the membranes were incubated overnight with primary antibodies of COL-2, SOX-9, ACAN and β-actin (Abcam, Cambridge, United Kingdom) at 4 °C, followed by incubating with HRP-labeled secondary antibodies. Finally, an electrochemiluminescence reagent autoradiography (Bio-Rad, Hercules, CA, United States) was used for scanning the images, and the bands were quantified by assessing the gray value with ImageJ. All of the data were normalized to multiples of β-actin. After 21 days of culturing, chondrogenic stainings were used to identify the formation of chondrocytes. In brief, after discarding the culture medium, the wells were washed with PBS for 3 times and followed by staining with alcian blue, safranin-O and toluidine blue according to the manufacturer’s instructions, respectively. An inverted microscope (ZEISS, Germany) was used for observing the distribution of chondrocytes, the area of positive staining was calculated for quantitative analysis.

### 2.8 Establishment of rat ACD model

In this study, 36 male SD rats (8 weeks old) with an average weight of 300–350 g were purchased from the Experimental Animal Center of Soochow University and the animal experiment was approved by the Ethics Committee of Soochow University. After anesthetizing with 4% pentobarbital sodium (25 mg/kg) intra-peritoneally, a 2 cm incision was made on the medial of knee and the patella was dislocated laterally to expose the knee joint. A cylindrical cartilage defect (2 mm in diameter and 1 mm in depth) was created on the condyles of femur with a tiny electric bone drill. For PBS group, 20 μL PBS solution was injected into the defect area; for KGN group, 20 μL of KGN solution was injected into the defect area; for KGN-NNPs group, 20 μL of KGN-NNPs solution was injected into the defect area. After the operation, the incision was sutured and 3 days of continuous injection of 200,000 U penicillin was given to prevent infection. At 6 and 12 weeks post-operation, the rats were sacrificed and their knee joints were harvested for further analysis.

### 2.9 Macroscopic assessment and histological analysis

Firstly, all specimens were evaluated macroscopically and quantified with the ICRS scoring system (van den Borne et al., 2007). A micro-CT scan system (Skyscan 1,176, Kontich, Belgium) was employed to evaluate the reconstruction of cartilage, bone and subchondral bone with a scanning parameter at 65 kV, 385 mA, 1 mm AI filter. Bone volume/Tissue volume (BV/TV) and trabecular thickness (Tb.Th) were used to quantitatively evaluate the osteochondral repair condition. Then, the samples were fixed with 4% paraformaldehyde for 24 h and followed by decalcifying with 10% EDTA for 1 month at room temperature. After gradient dehydration with different concentrations of ethanol, the samples were permeabilized with xylene for 10 min, embedded in paraffin and sectioned into 6 μm-thick slices. Then, hematoxylin and eosin (H&E) staining, immunohistochemical (IHC) staining for collagen-II (COL-II) and Aggrecan were performed. Wakitani scoring of histopathology was applied for quantitatively assess the repair of articular cartilage (Wakitani et al., 1994). Positive areas of COL-II and proteoglycans were also calculated for quantitative analysis.

### 2.10 Statistical analysis

All data in this work were presented as Mean ± Standard deviation (S.D.) and all statistical analyses were processed using one/two-way ANOVA and Turkey’s multiple comparison test with Graphpad Prism 10 software. In all datasets, *n* = 3 independent experiments were performed, and a *P* value <0.05 was considered to be statistically significant.

## 3 Results

### 3.1 Preparation and characterization of KGN-NNPs

**FIGURE 2:**
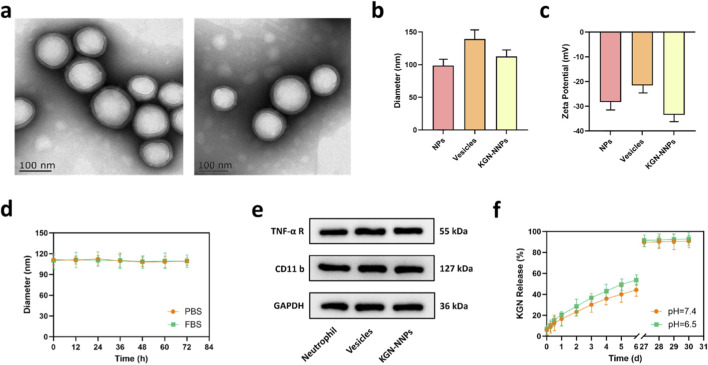
Preparation and characterization of KGN-NNPs. **(a)** Representative TEM images of KGN-NNPs (scale bars, 100 nm). **(b)** Particle sizes of PLGA NPs, neutrophil membrane vesicles and KGN-NNPs. **(c)** Zeta potentials of PLGA NPs, neutrophil membrane vesicles and KGN-NNPs. **(d)** Stability of KGN-NNPs in PBS and FBS by measuring particle size. **(e)** Western blot analysis for TNF-α R and CD11b protein expressions of neutrophil, vesicles and KGN-NNPs. **(f)** Drug release test of KGN-NNPs at pH = 7.4 and pH = 6.5. Data were presented as mean ± S.D. For particle size, zeta potential and drug release measurement, *n* = 3 independent tests were performed.

TEM showed that the synthesized KGN-NNPs had a typical shell-core structure, which was uniform in size and evenly dispersed in the medium (Figure 2a). DLS measurement revealed that the average diameter of KGN-NNPs was about 112.6 ± 1.8 nm, which was basically the same size as that observed by TEM (Figure 2b). The zeta potential of KGN-NNPs was about −33.5 ± 1.1 mV, indicating that the nanosystem was relatively stable and not prone to particle aggregation (Figure 2c). Subsequently, the KGN-NNPs maintained a stable particle size of about 109.5 ± 1.4 nm both in PBS and FBS for at least 72 h (Figure 2d). The encapsulation efficiency of KGN-NNPs measured by dialysis method was 76.7% ± 1.3%, and the drug loading capacity was 18.1% ± 0.6%. The results of WB presented uniform size and shape of bands for TNF-α R and CD11 b between neutrophil, vesicles and KGN-NNPs, which confirmed that the characteristic membrane protein receptors of neutrophil were well-preserved during the process of separating and coating (Figure 2e). The drug release ability of KGN-NNPs was tested under the environmental condition of pH = 7.4 and 6.5, respectively. Within the first week of the experiment, a phenomenon similar to “burst release” appeared in both sets of data, as the release rate of KGN reached almost 50% at 7 days, which was more obvious at the acidic condition of pH 6.5. With the extension of time, at about 1 month, the drug release rate of the two groups reached more than 90%, and was relatively close (Figure 2f).

### 3.2 *In vitro* biocompatibility of KGN-NNPs

**FIGURE 3:**
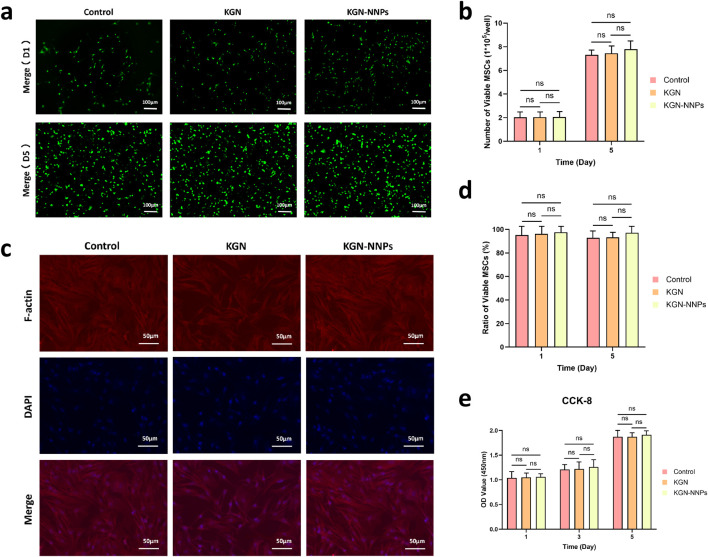
Biocompatibility evaluation of KGN-NNPs. **(a)** Live/Dead staining of MSCs at day 1 and day 5 after culturing with KGN and KGN-NNPs (scale bars, 100 μm). **(b)** Number of viable MSCs on day 1 and day 5 after culturing. **(c)** Cytoskeleton staining of MSCs at day 3 after culturing with KGN and KGN-NNPs (scale bars, 50 μm). **(d)** Ratio of viable MSCs on day 1 and day 5 after culturing. **(e)** Absorbance at 450 nm was detected at day 1, day 3 and day 5 after culturing MSCs with KGN and KGN-NNPs. Data were presented as mean ± S.D. Ns indicated no significance. In all datasets, *n* = 3 independent experiments were performed.

Live/Dead staining, CCK-8 assay and cytoskeleton staining were performed to ensure that the synthesized KGN-NNPs were not cytotoxic and suitable for cell culture. From the results of Live/Dead staining, it can be seen that the cell numbers in all groups were relatively low on day 1 of culturing (Figure 3a). As the culture time progressed, the cell numbers in all groups had significantly increased by day 5, from approximately 2 × 10^5^ cells/well to over 7 × 10^5^ cells/well. Moreover, there was no statistical difference between the groups, suggesting that KGN-NNPs had no significant effect on the proliferation of MSCs (Figure 3b). The KGN-NNPs group had similar number and proportion of viable cells to KGN group and Control group either on day 1 or day 5 with no significant difference (Figure 3d). CCK-8 assay was also applied for further confirming the *in vitro* safety of KGN-NNPs. There was no significant statistical difference in the absorbance value between Control group, KGN group and KGN-NNPs group on day 1, day 3 and day 5 after culturing (Figure 3e). The absorbance increased with the extension of culture time, which was consistent with the results of Live/Dead staining, suggesting that MSCs proliferated well in KGN-NNPs. The results of cytoskeleton staining showed that MSCs spread well after 3 days of culturing in all groups, and the morphology of F-actin was normal (Figure 3c). These above results suggest that KGN-NNPs had good biocompatibility and was suitable for MSCs survival and proliferation.

### 3.3 *In vitro* anti-inflammation capability of KGN-NNPs

**FIGURE 4:**
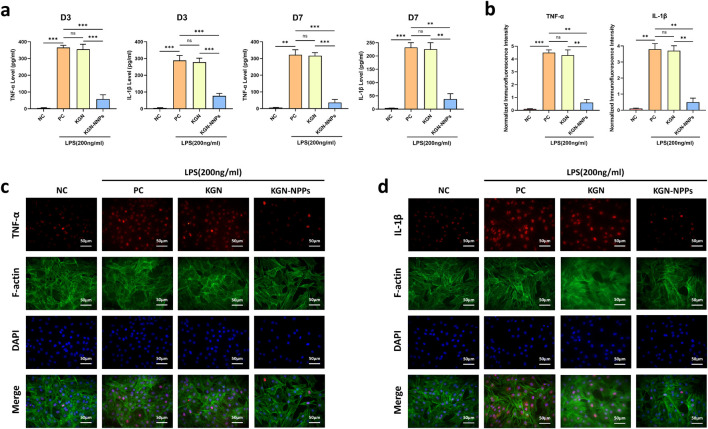
Assessment for *in vitro* anti-inflammation ability of KGN-NNPs. **(a)** ELISA tests for TNF-α and IL-1β after culturing MSCs with KGN and KGN-NNPs at day 3 and day 7. **(b)** Normalized immunofluorescence intensity for TNF-α and IL-1β after culturing MSCs with KGN and KGN-NNPs. **(c)** Immunofluorescence staining for TNF-α after culturing MSCs with KGN and KGN-NNPs (scale bars, 50 μm). **(d)** Immunofluorescence staining for IL-1β after culturing MSCs with KGN and KGN-NNPs (scale bars, 50 μm). Data were presented as mean ± S.D. **P < 0.01, ***P < 0.001, ns indicated no significance. In all datasets, *n* = 3 independent experiments were performed.

ELISA, immunofluorescence staining and qRT-PCR analysis were used to evaluate the *in vitro* anti-inflammatory ability of KGN-NNPs. On the day 3 after LPS induction, the TNF-α and IL-1β levels in the PC group had already increased significantly, which were significantly higher than those in the NC group, suggesting that an *in vitro* cellular inflammation model had been successfully established. The TNF-α and IL-1β levels of KGN group were similar to those of the PC group, and there was no significant difference between the two groups (P > 0.05); however, the TNF-α and IL-1β levels in the KGN-NNPs group were significantly lower than those in the PC and KGN group (P < 0.001). On the day 7 after LPS induction, the inflammatory factor levels in all groups had decreased to some extent, and the KGN-NNPs group was still significantly lower than the other two groups (P < 0.001), while there was no significant difference between the KGN group and the PC group (P > 0.05), which was similar to the results obtained on the day 3 (Figure 4a).

From the images of immunofluorescence staining, it can be seen that the expression of TNF-α and IL-1β in MSCs were very limited without adding LPS to the culture medium, the fluorescence intensity of both inflammatory factors were very weak (Figures 4c,d). While after the intervention of LPS, MSCs secreted a large amount of pro-inflammatory factors, and the fluorescence intensity increased significantly. In the KGN group, the fluorescence intensity of TNF-α and IL-1β were similar to that of the PC group, with no significant statistical difference (P > 0.05); however, the fluorescence intensity of these pro-inflammatory factors in the KGN-NNPs group were significantly lower than that of the PC group (P < 0.01) (Figure 4b). These results indicated that KGN-NNPs had a high efficiency in adsorbing inflammatory factors, participated in and mediated the negative regulation of intracellular inflammatory responses.

**FIGURE 5:**
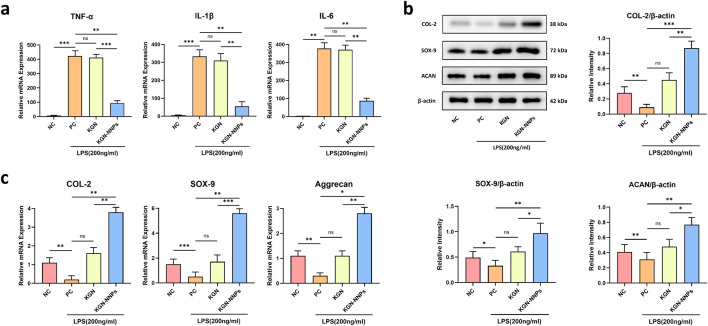
Assessment for *in vitro* anti-inflammation and chondrogenic abilities of KGN-NNPs. **(a)** qRT-PCR analysis for TNF-α, IL-1β and IL-6 after culturing MSCs with KGN and KGN-NNPs. **(b)** Quantitative Western blot analysis for COL-2, SOX-9 and ACAN after culturing MSCs with KGN and KGN-NNPs. **(c)** qRT-PCR analysis for COL-2, SOX-9 and Aggrecan after culturing MSCs with KGN and KGN-NNPs. Data were presented as mean ± S.D. *P < 0.05, **P < 0.01, ***P < 0.001, ns indicated no significance. In all datasets, *n* = 3 independent experiments were performed.

The expression levels of inflammation-related genes were further verified by qRT-PCR. In the absence of pro-inflammatory factors, the mRNA levels of TNF-α, IL-1β and IL-6 genes in MSCs were very low and almost undetectable. However, under the influence of LPS, their mRNA levels increased significantly, at least by more than 50 times. The mRNA levels of TNF-α, IL-1β, and IL-6 in the KGN group were slightly lower than those in the PC group, but there was no significant statistical difference between them (P > 0.05). The mRNA levels of these inflammatory factors in the KGN-NNPs group were significantly lower than those in the PC group (P < 0.01), especially for IL-1β (Figure 5a). The experimental results of qRT-PCR showed that KGN-NNPs inhibited the transcription of inflammation-related genes, reflecting its powerful ability in controlling and reducing the level of intracellelar inflammation.

### 3.4 *In vitro* chondrogenic induction ability of KGN-NNPs

The *in vitro* chondrogenic induction ability of KGN-NNPs was evaluated through qRT-PCR, WB and chondrogenic staining. After intervention with LPS for 24 h and culture with different groups of drugs for 7 days, the expression of cartilage-related genes including COL-2, SOX-9 and Aggrecan were further detected by qRT-PCR. Compared with the NC group, the relative mRNA expression levels of the genes were significantly lower in the PC group (P < 0.01); for KGN group, there was no significant statistical difference between KGN group and PC group (P > 0.05); the expression levels of all genes in the KGN-NNPs group were significantly higher than those in the PC group (P < 0.05), with COL-2 being 19.7 times higher, SOX-9 being 11.2 times higher, and Aggrecan being 9.3 times higher (Figure 5c). These results indicate that KGN-NNPs can better alleviate the effect of inflammation on the chondrogenic differentiation of MSCs, thereby promoting the gene expression of chondrocytes.

WB was also conducted to evaluate the chondrogenic capability of different groups after 7 days of culturing. Due to the impact of LPS intervention, the expression of targeting proteins including COL-2, SOX-9 and ACAN in PC group decreased obviously compared with NC group, which was proved by semi-quantitative analysis of bands (P < 0.05). The expression of these proteins in the KGN group were higher than that in the PC group, but there was no statistically significant difference between them (P > 0.05). The expression of targeting proteins in the KGN-NNPs group were the highest (P < 0.01), with COL-2 being 9.6 times that of the PC group, SOX-9 being 2.9 times that of the PC group, and ACAN being 2.5 times that of the PC group (Figure 5b). The results of WB were highly consistent with the results of qRT-PCR, indicating that KGN-NNPs could regulate the entire process of transcription and translation for chondrogenic gene expression, and thus significantly promote the differentiation of MSCs into chondrocytes.

**FIGURE 6:**
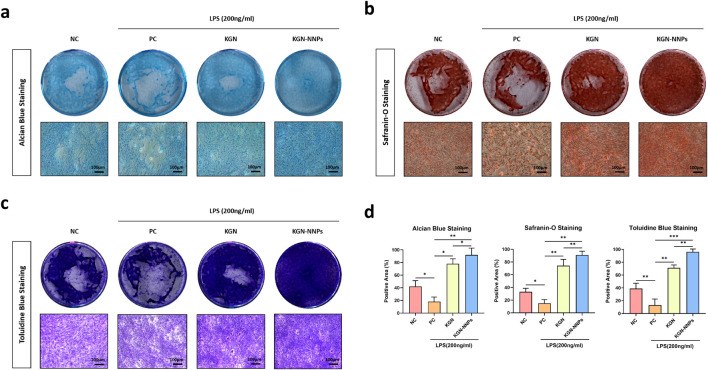
Assessment for *in vitro* chondrogenic ability of KGN-NNPs. **(a)** Alcian blue staining of MSCs after culturing with KGN and KGN-NNPs (scale bars, 100 μm). **(b)** Safranin-O staining of MSCs after culturing with KGN and KGN-NNPs (scale bars, 100 μm). **(c)** Toluidine blue staining of MSCs after culturing with KGN and KGN-NNPs (scale bars, 100 μm). **(d)** Quantitative analysis for the area of positive staining of MSCs after culturing with KGN and KGN-NNPs. Data were presented as mean ± S.D. *P < 0.05, **P < 0.01, ***P < 0.001. In all datasets, *n* = 3 independent experiments were performed.

After 21 days of culturing, classic chondrogenic staining methods incluidng alcian blue staining, safranin-O staining and toluidine blue staining were conducted to identify the formation of chondrocytes. The staining results showed that MSCs in NC group proliferated well due to the absence of inflammatory factors, and a large amount of cells could be observed (Figures 6a–c). In the PC group, due to the presence of LPS-induced inflammatory factors, the number of cells were significantly reduced, and there were more apoptotic cell nuclei. Meanwhile, the staining area and intensity were significantly less than those in the NC group (Figures 6a–d). Although under the influence of inflammatory mediators, KGN still played a certain role in promoting MSCs chondrogenesis, and the staining areas were larger than that of NC group and PC group, but still smaller than that of KGN-NNPs group (Figures 6a–d). The best result of chondrogenic differentiation staining was observed in the KGN-NNPs group, where a large number of chondrocytes could be observed and a dense ECM-like structure was formed, with the highest staining intensity, which suggesting that KGN-NNPs have both excellent anti-inflammatory function and chondrogenesis function (Figures 6a–d).

### 3.5 *In vivo* cartilage regenerative evaluation

**FIGURE 7:**
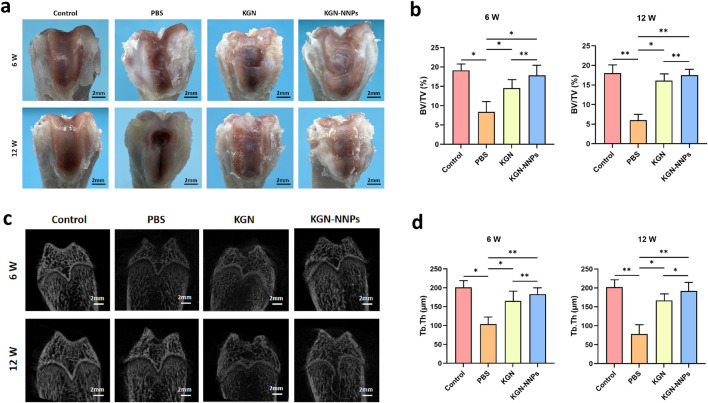
Assessment for *in vivo* chondrogenic ability of KGN-NNPs. **(a)** Macroscopic evaluation of cartilage repair in rat ACD model at 6 weeks and 12 weeks post-operation (scale bars, 2 mm). **(b)** BV/TV value of samples at 6 weeks and 12 weeks post-operation. **(c)** Micro-CT scanning of osteochondral repair for different groups at 6 weeks and 12 weeks post-operation (scale bars, 2 mm). **(d)** Tb.Th value of samples at 6 weeks and 12 weeks post-operation. Data were presented as mean ± S.D. *P < 0.05, **P < 0.01. In all datasets, *n* = 3 independent experiments were performed.

A rat ACD model was successfully established for investigating the *in vivo* repair effect of KGN-NNPs. From the appearance of the samples, at 6 weeks after operation, a moderate amount of cartilage tissue had been generated in the KGN-NNPs group, and the repair extent was significantly larger than that in the KGN group. At 12 weeks after operation, hyaline cartilage formation was observed in the KGN-NNPs group, and the edge was integrated with the surrounding tissue, while the edge was still clearly visible in the KGN group. The repair effect of the two groups were both better than that of the PBS group (Figure 7a). Micro-CT scanning showed that the KGN-NNPs group had the best osteochondral repair effect at both 6 and 12 weeks after operation, and the subchondral bone was almost completely reconstructed at 12 weeks after operation, while the osteochondral defects in PBS and KGN groups were still clearly visible (Figure 7c). The BV/TV and Tb.Th in the KGN-NNPs group were significantly higher than those in the PBS and KGN groups, and were close to the Control group at 12 weeks after surgery (Figures 7b,d).

**FIGURE 8:**
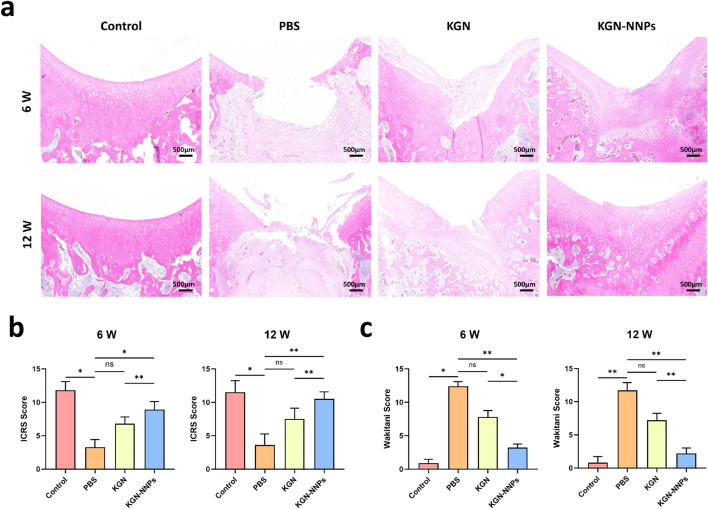
Assessment for *in vivo* chondrogenic ability of KGN-NNPs. **(a)** H&E staining of articular cartilage sections at 6 weeks and 12 weeks post-operation (scale bars, 500 μm). **(b)** ICRS scores of the histopathologic evaluation. **(c)** Wakitani score of the histopathologic evaluation. Data were presented as mean ± S.D. *P < 0.05, **P < 0.01, ns indicated no significance. In all datasets, *n* = 3 independent experiments were performed.

Further assessment of cartilage repair was conducted with H&E staining (Figure 8a). At 6 weeks post-operation, there was almost no new cartilage tissue in the PBS group; for KGN group, the cartilage defect was still quite noticeable, and the newly formed cartilage cells were arranged in a disorderly manner; for KGN-NNPs group, there were already many longitudinally arranged cartilage cells generated. At 12 weeks post-operation, the cartilage layer repair effect was ideal in the KGN-NNPs group, with the thickness basically restored to normal. However, the repair effect of KGN group was poor, with a depression still visible on the articular cartilage surface, suggesting insufficient amount of newly formed cartilage tissue. There was no progress in the repair of articular cartilage for PBS group compared with 6 weeks post-operation. ICRS scoring system and Wakitani scoring system were used to quantitatively evaluate the repair of ACD (Figures 8b,c). The ICRS score of KGN-NNPs group was 8.9 ± 0.4 at 6 weeks after surgery, which was better than 6.8 ± 0.3 of KGN group. At 12 weeks after operation, the ICRS score of KGN-NNPs group was 10.5 ± 0.6, which was close to the Control group (11.2 ± 0.5) and better than the KGN group (7.5 ± 0.8). Either at 6 weeks or 12 weeks post-operatively, the Wakitani score of the KGN-NNPs group was the lowest and closest to the Control group among the three invention groups, suggesting that the newly formed chondrogenic tissue in this group were closest to transparent cartilage, with a smooth surface and good fusion with normal cartilage tissue.

**FIGURE 9:**
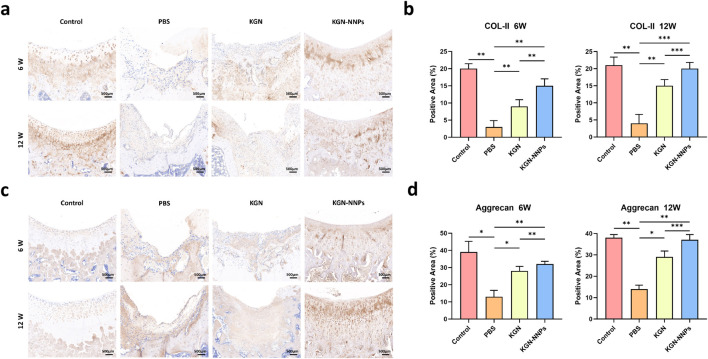
Assessment for *in vivo* chondrogenic ability of KGN-NNPs. **(a)** Immunohistochemical staining for COL-II of articular cartilage sections at 6 weeks and 12 weeks post-operation (scale bars, 500 μm). **(b)** Quantitative analysis of positive staining area for COL-II based on immunohistochemical staining. **(c)** Immunohistochemical staining for aggrecan of articular cartilage sections at 6 weeks and 12 weeks post-operation (scale bars, 500 μm). **(d)** Quantitative analysis of positive staining area for aggrecan based on immunohistochemical staining. Data were presented as mean ± S.D. *P < 0.05, **P < 0.01, ***P < 0.001. In all datasets, *n* = 3 independent experiments were performed.

Immunohistochemical staining was performed to determine the amount of COL-II and proteoglycans in the repaired tissue (Figures 9a,c). At 6 weeks post-operation, the cartilage defect was clearly visible in the PBS group, with no collagen filling it; for KGN group, in addition to the formation of chondrocytes, a small amount of COL-II and proteoglycans were also observed; for KGN-NNPs group, a large amount of COL-II was formed and deposited, and the amount of proteoglycans was significantly higher than that in KGN group. At 12 weeks post-operation, the deposition of COL-II was more obvious in the KGN-NNPs group, similar to the Control group, and the new chondrocytes were evenly distributed in the cartilage layer; the repair effect of the KGN group was similar to that at 6 weeks post-operation, with fewer chondrocytes and less obvious collagen deposition, forming abnormal cartilage tissue; no new chondrocytes or COL-II were seen in the PBS group (Figures 9a,c). Quantitative analysis showed that at 6 weeks after operation, the positive staining area of COL-II in KGN-NNPs group was 15.1% ± 1.8%, which was significantly higher than that of 9.6% ± 1.1% in KGN group and 3.5% ± 0.6% in PBS group. At 12 weeks after operation, the positive staining area in KGN-NNPs group was 20.8% ± 2.3%, which was still significantly higher than that of 14.6% ± 1.5% in KGN group and 4.2% ± 0.8% in PBS group (Figure 9b). The quantitative analysis of proteoglycans was similar to that of COL-II. At 6 weeks after surgery, the staining positive area in KGN-NNPs group was 32.5% ± 3.8%, while that was 27.6% ± 3.2% in KGN group and 13.1% ± 1.5% in PBS group. At 12 weeks after operation, the positive staining area of proteoglycans in KGN-NNPs group was 37.8 % ± 3.6%, which was significantly higher than that of 28.8% ± 2.9% in KGN group and 14.6% ± 1.7% in PBS group (Figure 9d). The above results indicated that KGN-NNPs had better cartilage repair effect *in vivo* compared with KGN group and PBS group.

## 4 Discussion

ACD has been receiving rising attention in the field of regenerative medicine due to its increasing incidence and inherent difficulty in self-healing. After cartilage damage, the release of local inflammatory factors accelerates the degradation of ECM and causes disorders in cell metabolism, making it extremely difficult to generate new chondrocytes (Shen et al., 2023; Klaymook et al., 2024). In this study, we designed and synthesized a novel neutrophil membrane-camouflaged PLGA NPs encapsulating KGN, and preliminarily evaluated its biocompatibility, anti-inflammatory capability and chondrogenic potential. To our delight, it exhibited targeted recognition and inhibition of inflammatory factors, excellent regulation of the inflammatory microenvironment, and remarkable promotion of cartilage repair in both *in vitro* and *in vivo* experiments.

Common modalities of NDDT include liposomes, micelles and polymers (Sun et al., 2020; Sun et al., 2021). As a kind of high polymer, PLGA was used as a drug carrier in the present study. Related experiments have proved its good biocompatibility and excellent degradative properties, which provided the possibility for sustained release of the encapsulated drugs and align with the long cycle of cartilage repair. According to literature, the repair time of bone tissue is generally up to about 3 months, while the repair time of cartilage tissue is usually longer due to poor blood supply. The retention time of conventional drugs after intra-articular injection is usually less than 1 week, which is far from meeting the repair conditions of bone or cartilage tissue. Benefit from the coating of PLGA, the KGN-NNPs prepared in this study could be released for more than 1 month, providing more drug action time for cartilage repair. In addition, previous studies indicate that the pH value of the local microenvironment after cartilage injury could drop to around 6.5, which is extremely unfavorable for cartilage regeneration (Zhang et al., 2020). Moreover, related studies have confirmed that the continuous high concentration of KGN plays a crucial role in the chondrogenic differentiation process of MSCs (Zhao et al., 2020; Almeida et al., 2020; Jing et al., 2019). In this study, through the coating of PLGA, a responsive release capability and a burst release of KGN in a short period could be achieved under an acidic environment, which was conducive to increasing the local concentration of KGN and providing favorable conditions for cartilage repair.

CMBT is one of the most novel technologies in the field of drug delivery (Hu et al., 2011). An important step in the implementation of the entire technology is the extraction and wrapping of the cell membrane, which plays a key role in maintaining the integrity of the cell membrane for subsequent research. Several methods for extracting cell membranes have been reported, such as cold precipitation, centrifugation, and ultrasonic method (Nica et al., 2023; Espinoza et al., 2023). From the perspective of biomedical engineering, hypotonic method is more suitable for lysing cells, followed by differential centrifugation to separate membrane components, and further purification by density gradient centrifugation (de Weerd et al., 2024; Zhu et al., 2023). In addition, the development of microfluidic homogenization technology in recent years is conducive to the realization of efficient and low-damage cell fragmentation, which provides a strong support for the industrial preparation of cell membranes (Xu et al., 2024). In this study, the cell membranes were carefully peeled off from neutrophils with ultrasound, and then wrapped onto the surface of PLGA NPs by co-extrusion. TEM showed that the core and shell diameter difference was 8.4 ± 0.5 nm, which was consistent with the single-layer thickness of neutrophil membrane reported in the literature as for 5–10 nm, indicating that the KGN-NNPs were prepared successfully. The WB analysis of receptor proteins on the surface of KGN-NNPs indicated that KGN-NNPs inherited important receptors including TNF-α and CD11b from the membrane of neutrophils, thus KGN-NNPs had the ability of responding to inflammation signals.

Benefit from the tiny size of NPs and the camouflage of the neutrophil membrane, KGN-NNPs could easily enter the cytosol of MSCs through endocytosis of the cell membrane and thus greatly enhanced the precision and efficiency of differentiation induction and inflammation control. Due to the retain of native membrane proteins, including key “self-recognition” markers such as CD 11b and CD 47, the KGN-NNPs avoided being cleared and phagocytized by the mononuclear phagocytic system (MPS) and macrophages during circulation in the body, thereby prolonging the circulation time. More importantly, the camouflage effect also reduces the probability of nonspecific endocytosis for KGN-NNPs by endothelial cells or cells at the site of inflammation (Zhang et al., 2018). After taking up by targeted cells, the natural properties of their membrane proteins may influence the endocytic pathway, favouring their entry into the cell by a non-lysosomal-dependent route, or slowing their transport to lysosomes to buy time for drug release in the cytosol (Hu et al., 2025). Meanwhile, the degradation process of PLGA could produce weakly acidic metabolites such as lactic acid and glycolic acid. When KGN-NNPs were ingested by cells via endocytosis and enter the acidic endosomal/lysosomal environment, degradation of PLGA could locally increase the acidity within the endosomal lumen (Su et al., 2021). The acidic environment would promote the protonation of the undegraded carboxyl groups on the PLGA chain, causing it to swell by water absorption and produce a “proton sponge effect” (Su et al., 2021; Singh and Singha, 2021). This process could disrupt the integrity of endosomal/lysosomal membranes, ultimately leading to membrane rupture, allowing the encapsulated KGN to be released into the cytoplasm rather than degraded by lysosomal hydrolases. As a result, KGN could diffuse efficiently in the cytoplasm and target its site of action through specific pathway (e.g., Smad 4/5 signaling pathway).

The progression of ACD is characterized by increased secretion of inflammatory factors, degradation of ECM and barrier for chondrogenic differentiation of MSCs (Sadri et al., 2023; Shi et al., 2022; Klabukov et al., 2023). The elevated levels of pro-inflammatory factors represented by TNF-α and IL-1β, accompanied by neutrophil infiltration, creating a local inflammatory microenvironment and accelerating the progression of cartilage degeneration (Subburaman and Edderkaoui, 2021). Therefore, down-regulating the expression of inflammatory factors, reducing immune cell infiltration, and restoring the decomposition and metabolic balance of ECM has become an important strategy for the treatment of ACD. In order to mimic the local inflammatory microenvironment of ACD, 200 ng/mL LPS was added to the culture medium in this study to induce cellular inflammation according to a previous research (Pearson et al., 2010). As a result, the KGN-NNPs synthesized in this study exhibited excellent ability of regulating immune microenvironment compared with KGN, which suggests that the coating of neutrophil membrane plays a key role in targeting adsorption of important pro-inflammatory cytokines including TNF-α and IL-1β. As a detection method with high sensitivity, specificity and good repeatability, ELISA is of great value for evaluating the progression of inflammation (Iria et al., 2022). By detecting the levels of inflammatory factors in the culture medium supernatant after LPS induction, the inflammation state in the extracellular environment could be sufficiently assessed, and the intracellular inflammation level could be indirectly reflected. Meanwhile, the intracellular inflammation level could be directly reflected through qRT-PCR analysis and WB. During the whole period of LPS intervention and cell culture, KGN-NNPs group consistently showed lower TNF-α and IL-1β levels, lower TNF-α, IL-1β, IL-6 gene and protein expression levels than KGN group and PC group, reflecting that KGN-NNPs could significantly downregulate the level of intracellular inflammation, showing its excellent anti-inflammatory properties.

The major component of articular cartilage is hyaline cartilage, which is composed of chondrocytes and ECM, while the ECM includes COL-II fibers, proteoglycans, and water (Tschaikowsky et al., 2022; Kilmer et al., 2020; Levinson et al., 2019). Therefore, COL-2 can be used as an important indicator to reflect the degree of collagen deposition and ECM degradation (Xie et al., 2021). In addition, the proteoglycan content in the ECM can be reflected by the expression of Aggrecan and ACAN (Hu et al., 2012; Wang C. et al., 2023). SOX-9 is a transcription factor that plays a key role in chondrocyte differentiation, and has been shown to upregulate the expression of COL-II in several studies (Jiang et al., 2019; Kim et al., 2013; Cha et al., 2013). In our study, the KGN-NNPs group significantly upregulated the expression of COL-2, SOX-9 and Aggrecan from the gene and protein dimensions respectively, even with the intervention of LPS. And finally, more compact cartilage ECM and more mature chondrocytes in the KGN-NNPs group were confirmed by staining, which indicated that KGN-NNPs not only have excellent anti-inflammatory ability, but also have strong chondrogenic ability. KGN is a potent inducer of chondrogenic differentiation for MSCs and has a potential role in the reconstruction of subchondral bone. In this present study, the method of neutrophil membrane camouflage and PLGA encapsulation not only achieved the effect of sustained release of KGN, maintained its long-term effective concentration, but also provided a protective effect to avoid immune clearance for KGN. In addition, the nanoscale size of KGN-NNPs makes it easier for KGN to penetrate into the damaged tissue and bind to endogenous MSCs. To our surprise, even when exogenous MSCs were not loaded in KGN-NNPs, excellent effect of cartilage regeneration was eventually achieved in the rat ACD model.

The study has certain limitations. Firstly, although the neutrophil membrane has not been reported to have the potential of osteochondral induction for MSCs, it is still meaningful to set up the group without the neutrophil membrane coating. The experimental grouping is expected to be further refined in the subsequent researches. Secondly, although the synthesized KGN-NNPs have shown good biocompatibility, outstanding anti-inflammatory and chondrogenic ability in basic research, there are several facts should be considered before the clinical application of such biomaterials. On the one hand, due to the special shape of cartilage defects, it is necessary to accurately locate the defect site before injection of such nanodrugs. On the other hand, the metabolic pathways of such nanodrugs in the circulation of human bodies need to be more clearly studied and clarified. In addition, the donor source of the cell membrane used to encase the nanodrugs is also an issue. In summary, cell membrane biomimetic nanoparticle is a promising strategy for drug delivery in the field of ACD repair, and further research need to be conducted in the future.

## 5 Conclusion

In this study, we designed and synthesized a novel injectable neutrophil membrane-camouflage nanoparticle with an inner core consisted of PLGA encapsulating KGN. The good biocompatibility, excellent ability of target-regulating inflammatory microenvironment, and strong capability of chondrogenic induction for KGN-NNPs were confirmed by *in vitro* experiments. Finally, it was applied to the rat ACD model and achieved satisfactory *in vivo* cartilage repair effect. The successful development of this cell membrane biomimetic nanodrug delivery system provides new ideas and strategies for the treatment of ACD.

## Data Availability

The raw data supporting the conclusions of this article will be made available by the authors, without undue reservation.
